# Neural Control of Voluntary Eye Closure: A Case Study and an fMRI Investigation of Blinking and Winking

**DOI:** 10.3233/BEN-2011-0355

**Published:** 2012-01-20

**Authors:** Martijn G. van Koningsbruggen, Marius V. Peelen, Eilir Davies, Robert D. Rafal

**Affiliations:** ^1^School of PsychologyBangor UniversityBangorUK; ^2^Center for Mind/Brain Sciences (CIMeC)University of TrentoRoveretoItaly

**Keywords:** Apraxia, stroke, winking, saccade, fMRI

## Abstract

The current paper describes a rare case of a patient who suffered from unilateral apraxia of eye closure as a result of a bilateral stroke. Interestingly, the patient’s ability to voluntarily close both eyelids (i.e. blinking) was not affected, indicating that different neural mechanisms control each type of eye closure. The stroke caused damage to a large part of the right frontal cortex, including the motor cortex, pre-motor cortex and the frontal eye field (FEF). The lesion in the left hemisphere was restricted to the FEF. In order to further study the neural mechanisms of eye closure, we conducted an fMRI study in a group of neurological healthy subjects. We found that all areas of the oculomotor cortex were activated by both left and right winking, including the FEF, supplementary eye field (SEF), and posterior parietal cortex (PPC). Blinking activated FEF and SEF, but not PPC. Both FEF and PPC were significantly more active during winking than blinking. Together, these results provide evidence for a critical role of the FEF in voluntary unilateral eye closure.

## 1. Introduction

Eye closure is achieved by co-ordinated inhibition of the levator palpebrae superioris by nuclei of the posterior commissure followed by reciprocal contraction of the orbicularis oculi [1]. Eye closure may be voluntary and sustained, or occur as brief reflexive blinks. There is evidence that different neural systems may be involved in voluntary and reflexive bilateral eye closure. For example, Gordon [2] compared the electromyographic features of reflexive bilateral blinks and sustained voluntary bilateral eye closure and observed motor units in the pretarsal region of the lid that were specifically activated during reflexive bilateral blinking. Moreover, Bell's phenomenon, an upward rotation of the globe that accompanies bilateral voluntarily eye closure, does not occur during bilateral reflexive blinks [3].

Few reports exist of patients in whom the ability to voluntarily close both their eyelids has been affected as a result of a *cortical* lesion. Most of these studies described patients with an inability to voluntarily close both eyes, known as apraxia of eye closure [4]. Patients with apraxia of eye closure have a normal bilateral blink reflex and normal bilateral eye closure during sleep, but when asked to close their eyes bilaterally they are unable to do so.

Colombo, Renzi and Gibertoni [4] studied a group of 51 stroke patients with contralateral gaze impairments, and five of these patients had apraxia of eye closure. All the patients reported in this study suffered from bilateral eye closure apraxia. The location and extent of the lesion in each patient were not specified, but it was mentioned that either the frontal and/or parietal lobe of the right hemisphere were affected. Miwa, Kigaohashi et al. [5] described a patient with a lesion in the right anterior cerebral artery territory, affecting the medial

ISSN 0953-4180/12/$27.50 © 2012 – IOS Press and the authors. All rights reserved frontal lobe, including the supplementary motor area. Initially, the patient could only close his ipsilesional eye when asked to voluntarily close both eyes. However, the ability to close both eyelids reflexively was not affected. Korn, Reith and Becker [6] reported a patient who, when asked to close both eyes, could not voluntarily close his left eye. The patient showed a normal bilateral blink reflex, and normal bilateral eye closure during sleep. He had suffered from a stroke in the right anterior cerebral artery territory that affected the frontal commissural fibres of the corpus callosum. The authors argued that the deficit was caused by the fact that the damage to the corpus callosum resulted in an uncoupling of the right motor system from the praxis centre in the left parietal cortex, resulting in a form of ideomotor apraxia.

Only one patient with an inability to voluntary close one eyelid while the other eyelid remains open (i.e., to wink) has been reported before. Belin, Larmande, Gaymard and Larmande [8] described a patient with a right parietal cortex lesion following tumour removal. Initially, the patient showed a form of motor impersistence in controlling his eyelids bilaterally, and as a result he could not keep his eyes voluntarily closed. After a month, the patient could again voluntarily close both eyes normally, but had developed an inability to only close one eye (i.e. winking). The patient was diagnosed with a unilateral eyelid closure apraxia of both eyes, since the disorder was caused by an inability to dissociate the two eyelid movements and was not caused by a motor deficit. The authors note that the initial motor impersistence and unilateral eyelid closure apraxia could be attributed to the same deficit.

The ability to independently control closure of either eye presumably developed because of the dazzle response. Emerging from dark or dim conditions into bright sunlight results in a 'dazzle response' – photophobia precipitating reflexive eye closure. Phenomenal photophobia is supra additive, being more than twice as great with two eyes open as with one. Plainis, Murray and Carden [7] measured the EMG activity of the extra-ocular muscles, a reliable measure of the dazzle response. After a three minute dark adaptation, a strong light source was presented either monocularly, or binocularly. They demonstrated that binocular stimulation led to a much higher EMG activity than the monocular stimulation. The ability to independently close one eye under these conditions reduces the duration of blindness accompanying the dazzle response, providing obvious survival advantage.

These few reports of patients with an impaired control of eyelid closure suggest that apraxia of eyelid closure can be caused by either damage to the primary or supplementary motor system [4,5], praxis centre of the parietal cortex [4,7,8], or a damage to the connection between these two areas [6]. Interestingly, these reports all involve patients with a right-sided lesion. The lack of any cases with left hemisphere lesion raises the question of whether there might be a right-sided dominance for voluntary eyelid control.

The current communication was motivated by observations in a patient who, after bilateral lesions of the frontal eye field (FEF), lost the ability to voluntarily close one eyelid independently from the other eyelid. We report the novel finding that a lesion of the FEF can cause apraxia of eyelid closure. The neural correlates of voluntary unilateral eye closure have not been studied before. Therefore, we also report observations from an fMRI investigation comparing voluntary unilateral (i.e. winking) and voluntary bilateral eye closure (i.e. blinking) in neurologically healthy individuals. This innovative approach makes it possible to contrast the findings of the case study with those of healthy individuals.

## 2. Case report

A 53-year-old woman was evaluated by the North Wales Brain Injury Service in 2003. Four years previously she had suffered from a cerebral haemorrhage in the right frontal lobe and a smaller haemorrhage to the left superior frontal lobe due to sagittal sinus thrombosus. She was initially comatose and awoke with dysphasia and left hemiplegia. 3D MRI scan (with 1mm sections) revealed a large, multi-loculated, cystic lesion involving much of the most dorsal part of the right frontal lobe, with a surrounding area of gliosis extending deep into the white matter and up to the ventricles. The right-sided lesion involves the hand area of the motor cortex, the frontal eye fields (FEF), pre-motor cortex, and the extensive parts of Brodman's area 9, 10, 45 and 46. There is, in addition, a smaller area of damage to the left frontal cortex, including part of the left FEF (see Fig. 1).

Motor examination revealed a spastic and non-functional left arm, but no sensory deficit. Neuropsychological assessment revealed impairments of executive function and working memory problems related to frontal lobe damage. There was also some decrease in verbal fluency, but no frank aphasia. Despite severe damage to her FEFs LB could still make eye-movements in all directions, and voluntarily blink bi-

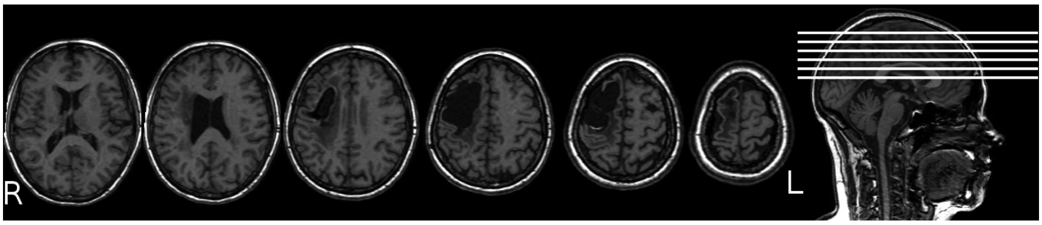

Fig. 1. T1-weighted normalised axial MRIcron showing lesions in patient LB, reconstructed using MRIcro software: The lines on the lateral view on the right indicate the levels of the axial sections. The right hemisphere lesion involves the hand area of the motor cortex, the frontal eye fields (FEF), pre-motor cortex, and the extensive parts of Brodman's area 9, 10, 45 and 46. There was, in addition, a small area of damage in the left frontal lobe, including part of the FEF.

laterally. She reported herself that she could no longer unilaterally close her left eye (i.e. wink), which she clearly remembered being able to do before the stroke (which was confirmed by her partner).

The patient's ability to voluntarily control her eyelids was assessed on two occasions, separated by more than a year. On both occasions she could bilaterally blink on verbal instructions, and keep both eyelids closed for a prolonged period of time ($>$ 30 seconds) when asked to do so. Involuntary lid closure in response to an approaching object was normal as well. Unilaterally closing her right eye was no problem either. However, she was unable to unilaterally close her left eye, which she recalls being able to do before her stroke. When both eyes were closed, she was unable to open her right eye without also opening the left eye, but she could open her left eye without opening her right eye.

In addition, an upper and lower face apraxia test was administered to test whether any other face-related apraxic disorders were present [9]. On both occasions, the patient could perform all the test items correctly, except one item of the upper face apraxia test: 'blink with your left eye'. Since she could execute all the other items correctly, her scores were well within the normal range, indicating that the apraxia was restricted to the unilateral eye closure. The apraxic disorder was still present a year after the first examination.

Only one patient with an inability to voluntarily close one eyelid (i.e. winking) has been reported before. This patient had a parietal lesion [8]. The currently examined patient had a large lesion affecting both the right and left frontal cortex, including the frontal oculomotor cortex on both sides. Interestingly, neither patient lost the ability to voluntarily blink bilaterally, suggesting that voluntary unilateral (i.e. winking) and bilateral eye closure (i.e. blinking) make use of at least partially different brain areas. In order to further explore the neural basis of winking and voluntary bilateral blinking we conducted an fMRI study.

## 3. fMRI study

The behavioural dissociation between voluntary bilateral blinking and unilateral winking observed in patient LB suggests that these two forms of eye closure are controlled, at least in part, by distinct neural mechanisms. The current fMRI study was aimed at revealing these neural mechanisms. Previous studies investigating neural mechanisms of voluntary bilateral blinking have found activation in frontal areas that are also activated during saccadic eye movements, including the FEF and supplementary eye fields (SEF) [10–14]. In contrast to frontal oculomotor cortex, parietal oculomotor cortex was not consistently activated during voluntary bilateral blinking in previous studies. The present study investigated the overlap between regions involved in voluntary bilateral eye closure (i.e. blinking), voluntary unilateral eye closure (i.e. winking), and voluntary eye movements.

## 4. Methods

### *4.1. Participants*

Five healthy adult volunteers (2 female) were recruited from the University of Wales, Bangor for the experiment. Participants satisfied all requirements in volunteer screening and gave informed consent. All participants were able to unilaterally close either eye. Ethics approval was obtained from the School of Psychology at the University of Wales, Bangor and the North-West Wales Health Trust. Participation was compensated at £20 per session.

### *4.2. Experimental paradigm*

Subjects performed 1 run of an eye movement task and 3 runs of an eye closure task.

The eye movement task consisted of 8 30-second blocks of eye movements and 8 30-second blocks of fixation (in an alternating fashion). At the start of each block, subjects were aurally instructed (through headphones) to either move their eyes ("move") or to fixate the center of a screen ("fixate"). No stimuli were presented on the screen throughout the experiment. Subjects were asked to make saccadic eye movements in the horizontal plane, moving from the center to the left, back to the center, to the right etc. at a pace of about one eye-movement every second. Parameters of the eye movements (e.g., amplitude, velocity) could not be recorded in the scanner.

The eye closure task consisted of the following conditions, each lasting 30 seconds: left winking, right winking, intentional bilateral blinking, and no blinking. These conditions were separated by 15 seconds fixation periods, which served as baseline in the statistical analyses. During the fixation periods, subjects were told to blink bilaterally as they would normally do. During the left and right winking conditions, subjects were asked to wink their left or right eye about twice per second. During the blinking condition, subjects were asked to voluntarily blink bilaterally about twice per second. During the no blinking condition, subjects were asked to inhibit any blink while keeping their eyes open. As subjects reported difficulties with this last condition, we did not further analyse these results. Similar to the eye movement task, subjects were aurally informed of the condition at the start of each block ("fixate", "left", "right", "both", "open", respectively). The order of experimental blocks was counterbalanced. Each of the 3 runs consisted of 9 fixation blocks and 2 blocks of each of the four conditions (left winking, right winking, intentional bilateral blinking, and no blinking).

### *4.3. Functional imaging and analysis*

#### *4.3.1. Data acquisition*

A 1.5T Philips MRI scanner with a SENSE parallel head coil was used. For functional imaging, a single-shot EPI sequence was used (TR = 3000 ms, TE = 50 ms, flip angle = 90°, FOV = 240, 30 axial slices, $64 \times 64$ in-plane matrix, 5 mm slice thickness). The scanned area covered the whole cortex and most of the cerebellum.

#### *4.3.2. Data analysis*

Pre-processing and statistical analysis of MRI data was performed using BrainVoyager 4.9 (Brain Innovation, Maastricht, The Netherlands). The first three volumes of each run were discarded in order to avoid differences in T1 saturation. Functional data were motion-corrected, low-frequency drifts were removed with a temporal high-pass filter (0.006 Hz), and the data were spatially smoothed with a Gaussian kernel (FWHM 6 mm). Functional data were manually co-registered with 3D anatomical T1 scans ($1 \times 1 \times 1.3$ mm resolution). The 3D scans were transformed into Talairach space, and the parameters for this transformation were subsequently applied to the co-registered functional data.

To generate predictors for the multiple-regression analyses, the event time series for each condition (e.g., the 30 s blocks) were convolved with a delayed gamma function (delta = 2.5 s; tau = 1.25 s) to model the hemodynamic response. Voxel time series were z-normalized for each run, and additional predictors accounting for baseline differences between runs were included in the design matrix.

## 5. Results

We contrasted each condition (eye movements, voluntary bilateral blinking, left winking, right winking) with a fixation baseline in group-average, fixed-effects whole-brain analyses.

Figure 2 shows the activation in the different conditions at a threshold of $p < 0.05$ (Bonferroni corrected for multiple comparisons). Replicating previous findings, eye movements strongly activated a bilateral fronto-parietal network, consisting of the frontal eye fields (FEF), posterior parietal cortex (PPC), and supplementary eye fields (SEF). The locations of these activations (see Table 1) were comparable to previous reports (e.g., [15]). Voluntary bilateral blinking significantly activated the FEF, but not the PPC or SEF. Interestingly, both left and right winking activated a fronto-parietal network that was very similar to the network involved in eye movements. No significant activations were observed when comparing left and right winking. A direct contrast between winking (averaged across left and right winking) minus voluntary bilateral blinking revealed significant ($p < 0.05$, corrected) greater bilateral activation in PPC and FEF during winking.

To assess the proximity of the eye closure activations to the areas activated by eye movements, we determined the Talairach coordinates of the peak activations (Table 1). The peaks of the activations in the voluntary unilateral (i.e. winking) and bilateral eye closure (i.e. blinking) tasks fell within a centimeter in any direction

Table 1
Talairach coordinates of activation peaks during eye movements, blinking, left winking and right winking. Blinking did not significantly activate the PPC and SEF in the whole-brain group analysis

| | Eye movements | | | Blinking | | | Left winking | | | Right winking | | |
|---|---|---|---|---|---|---|---|---|---|---|---|---|
| Region | x | y | z | x | y | z | x | y | z | x | y | z |
| Left FEF | −49 | −5 | 45 | −52 | −5 | 42 | −54 | −5 | 42 | −52 | −8 | 42 |
| Right FEF | 50 | −5 | 51 | 53 | −5 | 48 | 56 | −2 | 42 | 53 | −2 | 45 |
| Left PPC | −31 | −59 | 51 | −31 | −56 | 49 | −31 | −56 | 49 | | | |
| Right PPC | 32 | −44 | 48 | 29 | −44 | 48 | 29 | −44 | 45 | | | |
| SEF | −1 | −11 | −57 | −4 | −14 | 61 | −4 | −8 | 57 | | | |

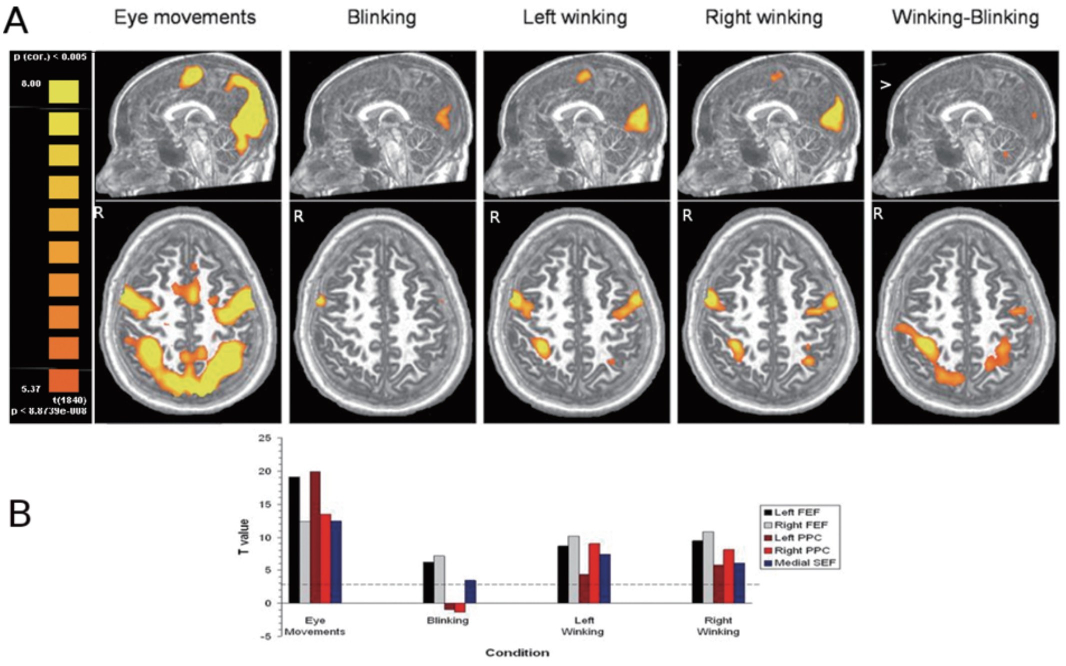

Fig. 2. Sagittal ($x = 0$; top row) and axial ($z = 48$; bottom row. See top right for the level of the axial cut) views of group activation (displayed at $p < 0.005$) during eye-movements, blinking, left winking, and right winking, versus a fixation baseline. Right panel shows the activation for the contrast between winking and blinking. Eye movements, left winking and right winking activated the FEF, PPC, SEF and visual cortex bilaterally, whereas blinking activated only the FEF and visual cortex bilaterally. Activations in the SEF were located medially, and extended into both hemispheres. Winking versus blinking activated the FEF and PPC bilaterally (activation in right FEF was located just inferior to the location of axial slice shown and is thus not visible in image). Left side of image depicts right side of brain. (B) T-values for each Region of Interest (Left FEF, Right FEF, Left PPC, Right PPC, and Medial SEF) for each Tasks. Dotted line indicates significance level.

(x, y, z) from the peaks in the eye movement task, indicating significant overlap of the eye closure activations and the eye movement activations.

Finally, we determined the T-values of activations in the eye closure conditions at the location of the eye movement related peak voxels (as reported in Table 1). This analysis was done to more directly investigate the possible involvement of eye movement related areas in voluntary eye closure. As can be seen in Fig. 2, significant activation in the FEF, PPC and SEF was found for the left and right winking conditions, confirming the whole-brain results. Again, significant activation in the left ($T = 6.4$, $p < 0.0001$) and right FEF ($T = 7.6$, $p < 0.0001$) was found for the bilateral blinking condition. Significant ($T = 3.5$, $p < 0.0005$) activation for voluntary bilateral blinking was found in the SEF that was not detected in the whole-brain analysis. We found again no evidence for activation in the PPC during voluntary bilateral blinking.

One explanation for the activation of eye movement related areas during winking could be that winking is (perhaps unconsciously) accompanied by eye movements. To test this, we recorded eye movements in 5 subjects outside the scanner during left and right winking (blocks of 30 seconds). No eye movements were detected during left and right winking (for recording methods see [16]), suggesting that the reported fMRI activation during winking was not due to co-occurring eye movements.

## 6. Discussion

We report a patient who lost the ability to unilaterally close her left eye (i.e. wink) after a large lesion in the right frontal lobe accompanied by a smaller lesion in left superior frontal cortex. She could still voluntarily blink with both eyes, suggesting that at least partly different cortical areas are involved in voluntary bilateral blinking and winking. This novel finding demonstrates that damage to the FEF can cause apraxia of unilateral eyelid closure. The fact that the patient had problems unilaterally closing her left eye could imply that this was mainly caused by the lesion to the FEF on the right side, which was much larger than the lesion in the left hemisphere. This is in accordance with a previous case report of patients with impaired voluntary eye closure. All those reports involve patients with a right-sided lesion.

The fMRI study investigated the neural correlates of voluntary unilateral eye closure (winking), voluntary bilateral eye closure (blinking), and saccadic eye movements. Blinking activated the FEF and SEF but not the PPC, replicating earlier studies [10–14]. Winking activated a fronto-parietal network that overlapped substantially with the network controlling eye movements (FEF, PPC, SEF), similarly for both left and right winking. Direct contrasts between winking and blinking revealed bilateral activation in FEF and PPC, suggesting a crucial role for both regions in the cortical control of winking.

Although none of the subjects reported that voluntary unilateral eye closure (i.e. winking) was more difficult than voluntary bilateral eye closure (i.e. blinking), the stronger activation in FEF and PPC for winking relative to blinking could in principle be caused by general differences in task difficulty, i.e. winking being a more difficult and less practiced movement than voluntary blinking, involving the coordinated control of both eyelids. This is a potential explanation for why the patient can still voluntarily blink, but can no longer voluntarily wink. If winking recruits the network more powerfully, damage to the network may lead to a bigger behavioural disruption. However, it is not clear why general task difficulty would result in the regionally specific increases we observed. Specifically, a task-difficulty account does not explain why both winking and eye movements, but not voluntary bilateral blinking, activated PPC. An alternative explanation is that winking (but not voluntary bilateral blinking) requires the execution of different motor plans for each eyelid, i.e. one eyelid has to remain open while the other must be closed. The motor, pre-motor, and parietal areas have been reported to be more active during anti-phasic compared to phasic bi-manual movements (for a review, see [17]). Importantly, the stronger activation in FEF and PPC for winking relative to voluntary bilateral blinking matches with the current and a previous [8] patient study that show that damage to either region can selectively impair winking.

In the fMRI study, the FEF was activated by both voluntary unilateral (winking) and bilateral (blinking) eye closure, with parts of the FEF being significantly more activated by winking than blinking. What could be the role of the FEF in winking? Winking involves the simultaneous control of both eyelids, with the active closure of one eyelid and the inhibition of the closure of the other eyelid. This coordinated control of both eyelids may be a function of the FEF, and may explain why left and right winking both activated left and right FEF to a similar extent. One possibility is thus that the right FEF controls the active closure of the left eye, while the left FEF simultaneously inhibits the closure of the right eye, and vice versa. Note, however, that patient LB was able to inhibit closing her left eye when asked to close her right eye. Furthermore, she could not inhibit *opening* her left eye when asked to open only her right eye. Thus inhibitory control of eye closure was most impaired in the eye contralesional to the hemisphere with complete destruction of the FEF.

The current fMRI study indicated that voluntary bilateral eye closure (i.e. blinking) in healthy subjects selectively activated the frontal regions of the oculomotor cortex (FEF and SEF), that were in large part damaged in patient LB. Yet, the patient was unimpaired in voluntary blinking. This suggests that the FEF may not be critically involved in voluntary blinking. We do not know if she lost the ability to blink voluntarily after the stroke and has since recovered this ability. It is possible that reorganizational processes may have occurred in the patient, allowing for a recovery of blinking. For example, Tombari et al. [18] examined patients with a motor hemiparesis using fMRI at three time points: immediately after the stroke, 4 months, and 12 months after the stroke. They found that compared to controls, other brain areas became active, which co-occurred with clinical improvements, suggesting that these areas took over part of the functions. Corbetta et al. [19] provided converging fMRI evidence for reorganizational processes in patients recovering from hemi-spatial neglect. In our patient, who was tested 3 years after the stroke, reorganizational processes are likely to have occurred. Thus, it could be possible that her ability to voluntarily blink recovered, but not her ability to wink, perhaps related to relatively more frequent blinking movements post stroke. However, the patient could not be tested on this task inside the MRI scanner, so we can only speculate. It would be interesting to test patients with similar lesions in the MRI scanner to directly study these re-organizational processes.

Schmidtke and Büttner-Ennever [20] reviewed the roles of sub-cortical and brainstem structures involved in the control of eyelids. However, there are almost no reports of patients with voluntary eyelid closure deficits resulting from *only* a subcortical lesion. Hirose et al. [21] described two patients who both had a small localised lesion in the basal ganglia. As a result of the lesion, both patients could no longer voluntary open their eyes. The authors hypothesized that the basal ganglia normally regulates cortical eye-opening systems through the thalamus, and that the absence of the basal ganglia causes disruption of the eye-opening system. The patient described here did not suffer from a sub-cortical lesion. Therefore, it is possible that subcortical areas could have been involved in the hypothesized reorganizational processes that occurred after the lesion.

In sum, we showed that in healthy controls voluntary bilateral eye closure (i.e. blinking) activates the FEF and SEF but not the PPC. Voluntary unilateral eye closure, in contrast, activates the FEF, SEF and PPC. These results fit with patient studies that show that both right parietal, and FEF lesions can impair unilateral eye closure. However, they do not explain why the patient could still voluntarily close both eyes, despite a bilateral lesion to the FEF. We suggest that reorganizational processes after the stroke could be a possible explanation for this difference.
